# Non-communicable diseases in Indian slums: re-framing the Social Determinants of Health

**DOI:** 10.1080/16549716.2018.1438840

**Published:** 2018-03-28

**Authors:** Lily Beth Lumagbas, Harry Laurence Selby Coleman, Joske Bunders, Antoine Pariente, Anne Belonje, Tjard de Cock Buning

**Affiliations:** a ILAW, Barangay Maybunga 1607, Pasig City, Philippines; b Athena Institute, Faculty of Science, Vrije Universiteit Amsterdam, 1081 HV Amsterdam, The Netherlands; c Le Centre de Recherche Inserm, University of Bordeaux Segalen, 33000 Bordeaux, France; d Dutch Heart Foundation, 2496 XD Den Haag, The Netherlands.

**Keywords:** Informal settlements, chronic illnesses, slum health, root cause analysis

## Abstract

**Background**: The epidemic of non-communicable diseases (NCDs) in slums has pushed its residents to heightened vulnerability. The Social Determinants of Health (SDH) framework has been used to understand the social dynamics and impact of NCDs, especially in poorly resourced communities. Whilst the SDH has helped to discredit the characterisation of NCDs as diseases of affluence, its impact on policy has been less definite. Given the multitude of factors that interact in the presentation of NCDs, operationalising the SDH for policies and programmes that account for the contextual complexity of slums has stalled.

**Objective**: To organise the complex networks of relations between SDH in slums so as to identify options for Indian municipal policy that are feasible to implement in the short term.

**Methods**: The study reviews the literature describing SDH in Indian slums, specifically those that establish causal relations between SDH and NCDs. Root cause analysis was then used to organise the identified relations of SDH and NCDs.

**Results**: Although poverty remains the largest structural determinant of health in slums, the multi-dimensional relations between SDH and NCDs are structured around four themes that describe the dynamics of slums, namely scarce clean water, low education, physical (in)activity and transportation. From the reviewed literature, four logic trees visualising the relations between SDH in slums and NCDs were constructed. The logic trees separate symptomatic problems from their more distal causes, and recommendations were formulated based on features of these relationships that are amenable to policy intervention.

**Conclusion**: Root cause analysis provides a means to focus the lens of examination of SDH, as evidenced here for Indian slums. It provides a guide for the development of policies that are grounded in the actual health concerns of people in slums, and takes account of the complex pathways through which diseases are socially constituted.

## Background

Of the 56 million deaths worldwide in 2012, non-communicable diseases (NCDs) accounted for 38 million (68%), and 28 (74%) million of these occurred in low- and middle-income countries (LMICs) [1]. There is growing empirical evidence that slums are locales of NCDs and their risk factors [2–5], whilst the absolute number of people living in slums increased from 767 million to 828 million from 2000 to 2010 in developing countries [6]. Continued slum formation and rises in NCD burden will place an increasing amount of the urban poor in chronic sickness or poverty. Understanding the causal pathways of NCD risk in slums, through distinguishing direct from indirect causes, is thus a key step to mitigating both national and international trends of NCD burden growth.

Slums can be defined by their physical and legal characteristics: ‘inadequate access to safe water; inadequate access to sanitation and other infrastructure; poor structural quality of housing; overcrowding; insecure residential status’ [6, p. 12]. The constraints to health placed on slum populations are unique given the combination of their urbanised lifestyle and limited access to healthcare, usually as a result of being relatively poor [7]. Whilst these shifts in lifestyle are often associated with economic development – such as, a higher consumption of processed foods and decreases in physical activity – they do not bring the benefits of greater access to healthcare provision [8]. Addressing NCDs in slums must be able to account for the wide-ranging causes of disease wrought by these contextual and social factors, and the Social Determinants of Health (SDH) offers one approach to studying this interplay.

Alongside the physiological causes of disease, the SDH provide an understanding of disease that incorporates the influence of social, behavioural, socio-economic and environmental factors [9]. The SDH includes education, income, transport, gender, age and social status, plus broader societal factors such as macroeconomic policy or cultural norms as influencers of disease [10], i.e., the causes of more evident risk factors such as physical inactivity or tobacco use. Its emphasis on the social conditions in which people live and work as determinants of health status make it relevant to an analysis of causal pathways of NCDs in slums.

The broader perspective on the epidemiological presentation of diseases given by the SDH has yielded a multitude of studies that substantiate the association between NCDs, their risk factors, and their socio-economic and environmental determinants. These studies have linked sedentary lifestyles and unhealthy eating patterns to obesity in LMICs [11], as well as the deleterious effects to health from low-quality education [12], poor built environment [13] and social interaction [14]. Empirical research taking the SDH as its lens has helped to discredit the characterisation of NCDs as diseases of affluence, yet its impact on policy is less definite. An analysis of the policy interventions taken by the Canadian government to improve health outcomes for the domestic type 2 diabetic population by Raphael et al. [15] found that public policy approaches targeting the SDH have been largely ineffectual. Indeed, the challenges associated with translating the SDH into local and national policies were highlighted in a review by Krumeich and Meershoek [16] who cautioned against the implementation of policies which fail to adapt the SDH based on features of the local context.

This raises the broad question on how the SDH can be used to identify options for policy, and, more specific to this study, how this can be done for the slum context. Slums and urbanisation were considered in the original report from the Commission on Social Determinants of Health [10], where slum upgrading was advocated as a part of their overarching recommendation to improve daily living conditions. This prioritised the provision of water and sanitation, electricity and paved streets for households irrespective of ability to pay. Further, the World Health Organization (WHO) Kobe Centre model for ‘Health in New Urban Settings’ [17] adapted the SDH into an ecological model that recognised urbanisation and the urban setting as influencers in health. This work emphasised governance as the critical causal pathway for addressing social determinants of health in urban settings, recommending that interventions be applied at the municipal level and comprise the following features: ecological and population-based that address multiple upstream determinants of health for entire communities; integrative in that multiple context-specific interventions can be applied simultaneously, and; systems-based and clearly linked to principles of good governance. The estimate in 2005 that global slum upgrading could be collectively financed by donors, local and national governments, and households for US$100 billion [18] typifies the recommendations that SDH work in slums has yielded; often broad and requiring large funding streams to implement. This study seeks to identify policy options for mitigating NCD risk in slums that are feasible for local governments, using an in-depth analysis of the causal relations of social determinants in slums.

**Figure 1.**
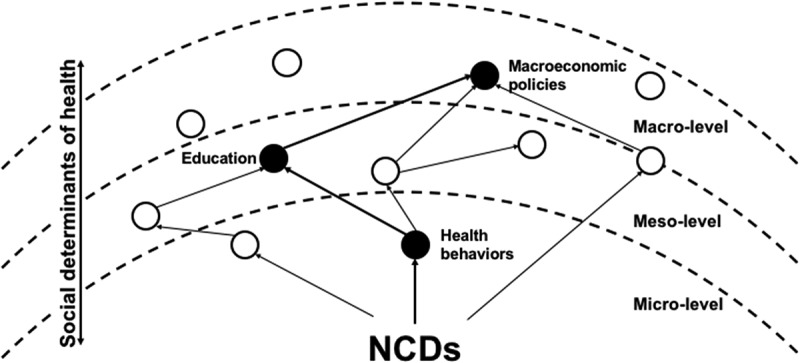
A schematic relation between the RCA and SDH. The RCA methodology is used to delineate cause and effect relationships between social determinants of NCDs in slums that have been empirically described in the literature. White circles represent empirically identified SDH of NCDs in slums, while the black circles represent a pathway of enhanced NCD risk in a slum individual, through the combination of social determinants.

Root cause analysis (RCA) encompasses a range of methodologies to scrutinise complex data by focusing on dependencies and relations [19]. Its main focus is to identify cause-and-effect relationships responsible for an adverse event or problem [20]. By reviewing the literature on the causes of NCDs in Indian slums from a systems perspective, this study looks to organise the complex networks of SDH in slums through RCA. Thus, the connection and triangulation of simple, linear causalities into larger causal trees illustrates in more detail the (documented) relations between SDH and NCDs in Indian slums. Based on empirical data of the reviewed literature, the logic trees visualise these relations and can be used to find causes at the intermediate level – between the symptoms of rising NCDs and intangible causes such as poverty – that are amenable to policy interventions [21]. There are some NCD risk factors well established by quantitative association (such as alcohol and tobacco use, and unhealthy diet) that haven’t been incorporated into the causal trees as the direction of their causilty in relation to other risk factors hasn’t been reported in the available and reliable literature. Whilst the SDH offer an all-ecompassing view of disease that recognise the impact of both local and distal factors to health, this perspective is often too expansive to discern concrete targets of interventions. RCA is suggested as one means to focus the lens of analysis in SDH studies to identify critical pathways of disease that policies can target, specifically at the local level (Figure 1).

## Methods

A narrative review of the literature was conducted to identify peer-reviewed papers that identify causal relationships between social determinants and NCDs in Indian slums. A broad range of search terms and databases was used with the aim of identifying literature in multiple disciplines, as opposed to those solely in the public health domain. Any combination of the following search terms was used to generate results; slums; informal settlements; non-communicable diseases; chronic illness; social determinants of health; socioeconomic status; urbanisation; health; illness; low- and middle-income countries; globalisation; poverty; social exclusion; and women. Searches were conducted in the following bibliographic databases: Academic Source Complete; JSTOR; CINAHL; ERIC; MEDLINE; PUBMED; Science Direct; Web of Science; and Humanities International Complete. Reference lists of relevant articles were also searched to identify additional literature.

The titles and abstracts of articles were reviewed and those deemed relevant were stored in a bibliographic database. The PDF files of included articles were sought before a full-text reading – applying the following inclusion and exclusion criteria – generated the final inventory of articles. Articles had to be published in English between 2000 and 2016, and use qualitative, quantitative or mixed methods to identify causal links between SDH and NCDs. Conceptual analyses of NCDs in Indian slums and SDH were also included. The following document types were accepted: peer-reviewed scientific articles, books, policy documents and government publications. Editorials, opinion pieces, position papers, pamphlets and monographs were excluded. Articles published in peer-reviewed scientific journals were primarily sought; however, some institutional reports – such as, from the WHO – were used if referenced in peer-reviewed publications. The relevance of articles was first judged by their reference to Indian slums, before seeking a description of the causal pathways of NCDs. As articles had to establish causal relations, quantitative papers had to take repeated measurements over time or incorporate a qualitative methodology to identify the direction of associations.

Root cause analysis (RCA) was used to organise the causal relations identified by reviewed literature into logic trees. RCA attempts to sort symptomatic problems from their deeper causes [21], and this study sought to do this through organizing the causal relations of NCDs in Indian slums that have been empirically identified in the literature. The included studies were inductively analysed, identifying recurrent themes before iteratively refining these as more literature was reviewed. Due to the constraints imposed by the search criteria and the focus of included research articles, the inductive generation of themes was limited by selection bias, which may underpin their strong correlation to typical characteristics of slums. These themes were: lack of clean water, education, physical (in)activity and transportation. Social determinants of NCDs were structured into four logic trees using these themes. The evidence from reviewed literature as it relates to the social determinants of NCDs in slums will be discussed per theme below.

## Results

From the inductive analysis of reviewed articles, four themes emerged that were used during the RCA to structure the social determinants of NCDs identified in slums: lack of clean water, education, physical (in)activity and transportation. Poverty has been cited as the most dominant structural determinant of health affecting individuals living in slums [17], yet rather than structuring the causal trees with poverty as the most deep-rooted cause, the analysis has sought to identify the consequences of poverty, or lack of income, that would generate opportunities for policy other than social protection policies or money transfers. Three types of literature data were used in the analysis, describing:

1. The presence of NCDs in slums (quantitative descriptions).
2. The connection between location and NCDs (quantitative and explanatory).
3. The experiences of people with NCDs in slums (qualitative and explanatory).

The following sections detail the results of the RCA of reviewed literature that delineates the causal pathways of NCDs in slums.

### Lack of clean water

In a broad literature review on health inequity in Asia, Friel et al. [22] stressed that the health of people in Asia is shaped by both socioe-conomic and environmental conditions, as well as persistent health inequities. They found that a lack of clean drinking water was a strong indicator of health inequity in Asia. One factor in the dearth of clean water in slums can be its contamination, such as by vectors, that raises NCD risk. This causal pathway was reported by Ogoina and Onyemelukwe [23] in their review, which linked the contamination of water by vectors to higher infectious disease prevalence, that leads to increased susceptibility to autoimmune diseases [24].

In India, socio-political and economic factors such as the lack of sanitation and poor water management are primary causes behind the country’s general lack of clean water. Following an extensive literature review and five regional consultations of key stakeholders, Cronin et al. [25] observed that the level of water pollution in India is critical: it can cause severe harm to people if left unattended, especially in the slums. Hence, sanitation, access to clean water, and its sustainability are India’s one of the most urgent concerns. The comparative case study of five cities by Allen et al. [26] showed that the inadequacy of public policies and private initiatives to combat the lack of clean water results in higher water prices for the peri-urban poor, which encourages them to use alternate means to access water. Anand’s study [27] in the slums of Mumbai expanded this notion, stating that slum dwellers’ access to water depends on complex cultural, political and empirical factors.

In a comparative study using the Indian Census of 2001, Goli et al. [28] noted that the lack of safe drinking water in Indian cities, particualrly in slums, serves as a marker for the poor living conditions of residents that lead to poor health. In their own comparative, ethnographic study covering slums in Chittagong, Dhaka, Hyderabad and Nairobi, Joshi et al. [29] emphasised that the focus on drinking water simplifies socio-economic and cultural complexities, as water is also used for laundry, bathing, cooking, personal hygiene and in domestic tasks. Reddy and Snehalatha [30] asserted that lacking clean water places slum women at a further disadvantage, as it is the women’s task to collect the water for the family and keep their surrounding area clean. This demonstrates the influence of cultural norms on water inequality, of which poverty, caste and gender are all factors. In his longitudinal study, Joshi [31] observed that women were ascribed the responsibility of water-related chores, summarising this as: ‘a good woman is one who performs these tasks which includes carrying and using water in the home’ (p. 57). As such, men are not expected to perform water tasks; doing so would be a loss of face.

Sahoo et al. [32] conducted grounded theory life-course research studying the impact of lacking sanitation on the lives of slum women in Odisha. The results of 56 in-depth interviews showed that sanitation far extends beyond defecation and urination, as many actions are performed by women to secure water for the entire family, such as carrying water, queueing for water and walking long distances. The ignorance of these tasks hides their heightened risk of accidents, violence and other dangers when they carry water or travel for bathing and other personal hygiene tasks. Further, newly married women and expectant mothers face the greatest burden by the lack of water and sanitation as compared to other women. To facilitate their entry into the community and to cope with the frequent urination during pregnancy, both respectively decrease their intake of water and food. A qualitative study by Khanna and Das [33] in rural Uttar Pradesh highlighted similar findings, observing that some women had to walk for hours to find water or a suitable place for personal hygiene, which puts them at risk of violence, physical overexertion, accidents and illnesses. They also noted that whilst women were responsible for securing water for the entire family, they had the lowest priority regarding personal use of water. Other studies have arrived at a similar conclusion [27,29,30].

Mahon and Fernandes [34] argued that the menstrual hygiene of women and girls is a neglected issue within water, sanitation and hygiene (WASH) in slums. Using the experiences of WaterAid in Nepal and India for their case study, they identified the religious and cultural taboos that fuel negative beliefs and behaviours towards menstruation in the region, e.g. menstruation is dirty, women’s impurity during menstruation, lack of awareness of the biological processes of menstruation, which significantly contributes to its neglect. This neglect can lead to illnesses such as menstrual infection, white discharge, itching, burning, ovarian swelling and frequent urination. During menstruation, women and girls experience more difficulties as they collect water – which they are not allowed to touch and use during menstruation – and perform personal hygiene. They worry about how and where to wash the clothes they use, and experience physical pain and shame as they attempt to gather water for the family.

**Figure 2.**
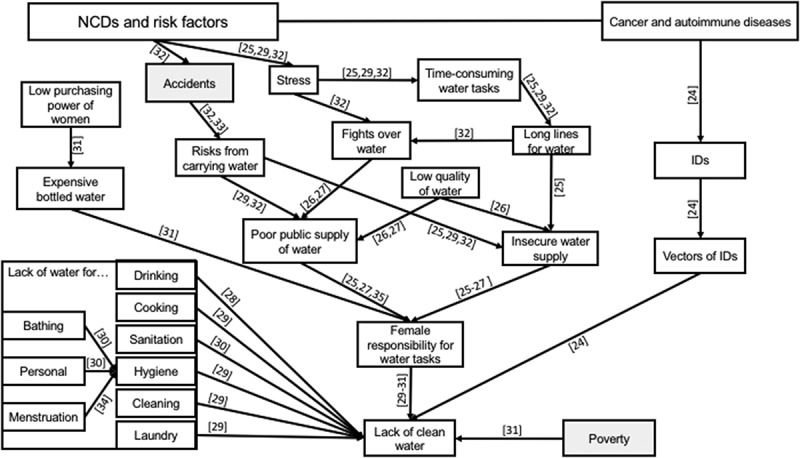
Logic tree structured around the lack of clean water in slums. Numbers in square brackets relate to the reference numbers of the literature list evidencing the causal direction of the relationship shown. The direction of arrows indicates deeper causes, lines without arrows highlight relations between concepts (usually one being a subset of the other), and shaded boxes indicate connecting determinants in the other figures.

Mehta [35] stated that the scarcity of clean water should also be connected to the wider socio-economic, political and cultural conditions of India. Institutionalised and state-supported strategies against water scarcity were set to benefit rich farmers and agro-industrial lobbyists more than the poorer groups, such as pastoralists and dryland cultivators, they were claiming to help. The relations between causal pathways of NCD risk and clean water scarcity are depicted in Figure 2.

### Education

A low level of education is one of the social determinants identified as a critical risk factor for developing CVD among women. In 2011, Yusuf et al. [36] performed a cohort study in Mumbai with the aim of determining the influence of education on CVD mortality. They found that CVD was the leading cause of death in both men and women across all levels of education; however, they observed it was highest amongst males and females who were illiterate or amongst those who had attended primary school only. The hazard ratio (HR) for both sexes was equivalent, whilst all other educational levels (middle school, secondary education and college) had a decreasing HR. They attributed their findings to the fact that better educated people have more access to information and more awareness of the issues surrounding NCD risk.

In a review of the literature regarding the education of children living in poverty, (10) Nambisan [37] noted the questionable quality of public schools on the one hand and exorbitant tuition fees of private schools on the other, which served to push children living in chronic poverty into further disadvantage. Using qualitative surveys of 417 households in two Delhi slums and reports from National Sample Surveys, Tsujita [38] analysed the factors affecting education deprivation of slum children in Delhi. She maintained that the inability of parents to pay for schooling (e.g. textbooks, examinations, tuition) combined with their negative perceptions towards education serve as a primary cause behind the high drop-out or absenteeism of slum children between the age of 5–14. Chugh [39] also studied factors behind student drop-out in slum students aged between 15 and 19, showing that gender, institutional limitations and parents’ negative perceptions of education as significant determinants. Purposively sampling 432 Delhi slum students who had dropped out, she found that, compared with boys, more girls were dropping out of school at an earlier age, often as they are asked to help their mothers with household chores or to care for their younger siblings [39]. Early marriage was also cited as a factor in girls dropping out, whilst boys would dropout from school to work. The study also highlighted that the quality of girls’ education is inferior to boys’, resulting from cultural beliefs of women’s limited contributions to family income. Conversely, boys’ education is prioritised as they are charged with caring for their parents. Wu et al. [40] substantiated the influence of culture on education, linking girls’ assignment to household chores, caring for siblings and marriage, to parents’ low prioritisation of female education.

In their survey of schools within the slums of East Delhi, Tooley and Dixon [41] also observed boys’ preferential education for boys. Further, they noted that whilst slums are often situated near city centres, many slum students have to walk 2–3 hours to reach their school as these charge less compared with inner-city schools. The long walks also serve to discourage attendance, especially amongst girls who cite the physical exhaustion and fears for their safety. Alarmed by the continuing increase in dropout rate amongst Indian primary school students, Sajjad et al. [42] conducted an in-depth study involving 129 students who had dropped out between 2010 and 2011. They found that despite the government’s efforts to provide universal education, the beliefs, values and traditions that discriminate against girls’ education and demand for boys to seek work were primary reasons for children dropping out. Explaining parent’s role in school drop-out rates, Mukherjee and Das [43] found that parents’ educational and work status, particularly the father’s, have a significant impact on children’s schooling and the pressure they experience to enter the work force. Dutt [44] argued that although girls’ education is their ticket to freedom, it is limited by cultural norms. She found that many social and cultural structures in India do not favour the pursuit of girls’ education, such as early marriage, their assignment to household chores, harassment in public spaces and poverty.

**Figure 3.**
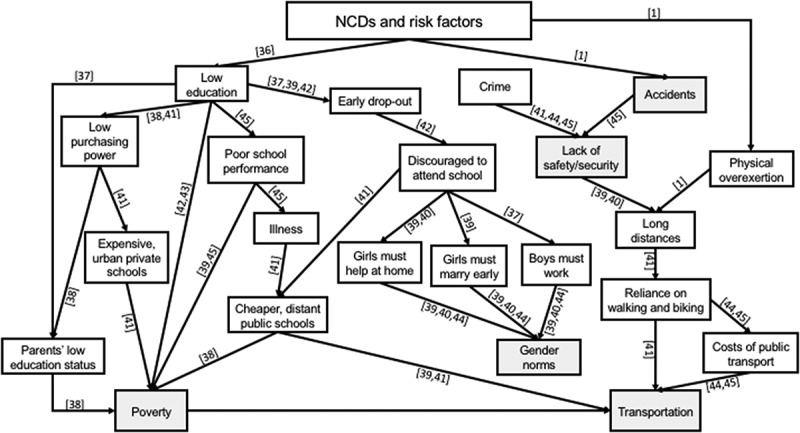
Logic tree structured around low education in the slums. Numbers in square brackets relate to the reference numbers of the literature list evidencing the causal direction of the relationship shown. The direction of arrows indicates deeper causes, lines without arrows highlight relations between concepts (usually one being a subset of the other), and shaded boxes indicate connecting determinants in the other figures.

Teachers’ perceptions and attitudes towards students have also been found to affect both male and female students’ performance [39]. Corporal punishments, discriminatory school environments, humiliation, insults and the abusive attitudes of teachers towards slum students all serve to discourage them from continuing their studies. Additionally, Mohanty’s [45] literature review and household survey in the slums of Lucknow and Kanpur concluded that originating from a scheduled caste, tribe or being a slum dweller increased the risk of low education. Figure 3 shows how the low education of slum residents as an NCD risk factor relates to opportunities that are limited by various socio-cultural and economic factors. These limited opportunities are further compounded by other factors such as age, limited awareness, limited accessibility, susceptibility to accidents and chronic physical pain [1].

### Physical (in)activity

The WHO [17] stated in its NCD report that physical activity (PA) is one of the most effective approaches to preventing NCDs, particularly CVD. The practice of this health behaviour, however, was linked by Mendelbaum [46] during his extensive ethnographic study of India to the social expectations imposed upon Indian boys, girls, men and women. Supporting this, Anand et al. [4] found through a quantitative survey of the slums in Faridabad that physical inactivity was higher amongst men than women. Tripathy et al. [47] used the STEPwise approach to NCD risk factor surveillance (STEPS) survey to draw a similar conclusion, citing the lack of recreational resources for PA amongst women in slums. The STEPS survey however, has been criticised for its inability to capture the physical activity related to housework, for which women are primarily responsible [4]. A cross-sectional study from Chomitz’ et al. [48] linked the cultural context of India that assigns women to household chores to their reportedly low PA. In their study of the association between built environment (BE) and PA, Adlakha et al. [49] supported the notion that the understanding of PA is limited to three categories of work, leisure and travel, which ignores the household work of people in slums. Manjrekar et al. [50] linked the lower PA of women in slums to a higher risk of developing NCDs in their comparative study between working and non-working women in urban India.

**Figure 4.**
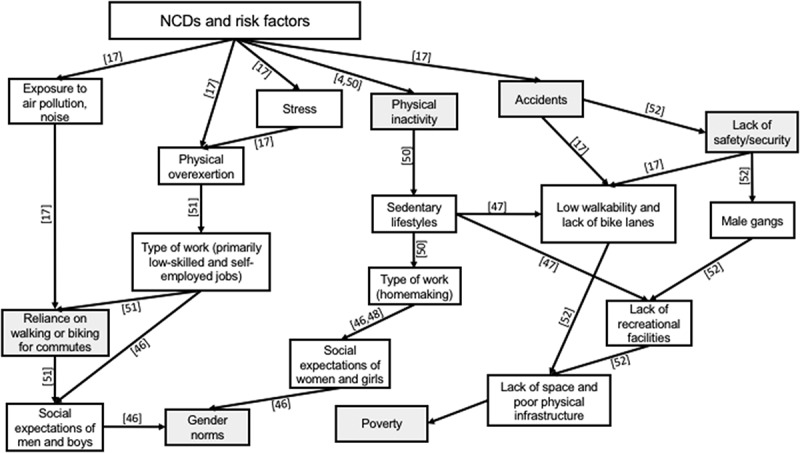
Logic tree structured around physical (in)activity. Numbers in square brackets relate to the reference numbers of the literature list evidencing the causal direction of the relationship shown. The direction of arrows indicates deeper causes, lines without arrows highlight relations between concepts (usually one being a subset of the other), and shaded boxes indicate connecting determinants in the other figures.

The issue of physical inactivity in the slums is due to many factors, such as the lack of green parks or safe spaces where people can walk and exercise to the cultural restrictions on women’s mobility, their burden of household chores, the lack of streetlights, and safety and security issues [17]. Similarly, whilst transportation is accessible in many slum communities due to their proximity to urban centres, few utilise it due to its high cost and opt to walk or bike for long hours instead. Within this context, many observations have been made about PA and slum dwellers. In studying the effect of BE on the PA of citizens of South India, Adlakha et al. [51] concluded that people with lower socio-economic status (SES) and living in an area of lower walkability spend more time walking (225 minutes/week) and biking (75 minutes/week) than people with higher SES in an area of high walkability (walking 30 mins/week; biking 1.0 min/week). They attributed the discrepancy to the prohibitive traffic conditions in Chennai, which discourage people with high SES from using their private vehicles. Low PA in slums has also been linked to safety and security issues. The WHO cited the lack of recreational facilities, and poor urban infrastructure of slums in general, as conditions for gang culture, primarily amongst men [52]. The effects of physical (in)activity on social determinants of NCD risk are depicted in Figure 4.

### Transportation

The WHO [52] report on social determinants for health equity in urban settings suggested urbanisation is a determinant of health. In an exploratory analysis of urban health, Mullen et al. [53] concluded in their *World Bank Report 2016* that while urbanisation brings with it certain health advantages, health disadvantages can also arise. In their case study in Chennai, Bhubanewaar, Meerut and Shillong, they noted that transportation is a critical factor in urban health, presenting both positive and negative opportunities.

Giles-Corti et al. [54] conducted a narrative literature review from various disciplines to study the connection between health and city planning. Although they concluded that the evidence in LMICs is limited to establish that urban and transport integration are critical for reducing health inequities, they found that traffic congestion is strongly associated with CVD. Likewise, undeveloped public transport and the frequent use of two-wheel motorcycles for mobility contribute to an increased risk of type 2 diabetes, obesity and hypertension due to reduced physical activity. Hence, they urged for greater collaboration between government agencies to deal with changing patterns in urban transportation. Adlakha et al. [49] examined the connection between BE and PA in Chennai, India, distributing the International Physical Activity and Environment Network (IPEN) survey questionnaire to 292 participants from four different SES. They concluded that people with a lower SES obtain their PA from travel (walking and biking), while people with a higher SES obtain their PA from leisure. As one of the first studies conducted on this issue in India, the authors recommended that their findings should be contextualised. In another study, Adlakha et al. [51] combined the IPEN with the Neighborhood Environment Walkability Scale (NEWS) to measure the effects of BE on PA. They observed that residents from both lower and higher SES meet the WHO’s recommended levels of PA, yet residents from lower SES get their PA from travel, whilst residents with higher SES obtain theirs from leisure and recreation. They also stated that public transportation is necessary for residents from lower SES as they cannot afford the price of an automobile to enhance mobility.

**Figure 5.**
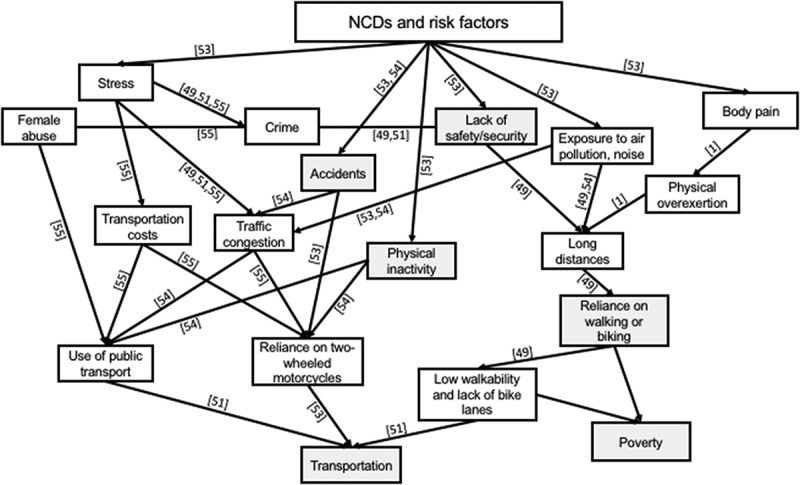
Logic tree structured around transportation in slums. Numbers in square brackets relate to the reference numbers of the literature list evidencing the causal direction of the relationship shown. The direction of arrows indicates deeper causes, lines without arrows highlight relations between concepts (usually one being a subset of the other), and shaded boxes indicate connecting determinants in the other figures.

In a case study of the impact of urbanisation on three Indian cities, Viswanath et al. [55] found that extant public transportation is unsustainable for several reasons. First, it is unaffordable: the cost of public transportation in Mumbai is twice that of London and five times that of New York. Second, it is inefficient, as ‘Delhi’s Metro can accommodate 6,520 rider per system km while Mexico’s 19,200, Moscow’s 21,400 and Sao Paulo’s 27,800’ (p. 64). Further, public transportation is often poorly integrated and out-of-date: the lack of feeder services minimise the scope for high ridership. Thus, transportation in urban settings shows how the arrangement of cities can influence the health and life of slum dwellers. Poor urban planning can aggravate slum conditions as it fails to account for the constraints imposed upon slum residents in their mobility. An overview of the relationship between transportation and NCD risk is visualised in Figure 5.

## Discussion

The commingling of risk factor pathways in slums combined with slum dwellers’ contextual responses create dynamic and complex interactions, which highlight the difficulty of establishing simple causal relations between separate determinants and NCDs. Male slum dwellers move in an open system, whilst women and girls are confined to a closed system within the unique conditions of the slums. The individual behaviours of slum residents in their environment, which ‘open up’ causal pathways of NCDs, cannot be tackled by policy recommendations based on lumped quantitative data, as this ignores the crucial interplay of the SDH in slum conditions, as well as culture, politics, poverty, economics and actions.

In analysing and reconstructing the existing literature on the SDH in Indian slums, the causal relations of NCD risk experienced by slum residents could be delineated. For example, by analysing the connection between the low education of women and NCDs, the RCA could infer how low education becomes an indirect determinant for a wider network of consequences that have an impact on health. More significantly, low education, on its own, is not a sufficient condition to develop NCDs and their risk factors. However, if it is combined with other elements that are present in slums, e.g. seclusion of women, the abusive attitude of teachers, low walkability, high costs of public transport, lack of clean water, it becomes a strong confounding factor increasing the risk of developing NCDs, particularly CVD. In this regard, the research extends many quantitative studies that have sought to characterise risk factor associations. For example, in Menon et al. [56], their quantitative survey on the prevalence of NCDs in poor rural settings of Kerala found that living below the poverty line (BPL) in a rural setting is associated with stroke (OR 1.33,1.04–1.69; p = 0.02) and COPD (OR 1.23, 1.15–1.32; p < 0.0001). Figures 2 and 3 of the RCA show that in slum settings, poverty interacts with many other contextually bound factors, such as the low education status of parents or the assignment of women to water tasks, which combine to produce a higher risk of developing NCDs.

Several conclusions can be drawn from this. First, a reframing of NCDs in slum settings is required. By considering slums as living spaces and communities, it dispels the assumption that slums refer to both the people and their condition as almost synonymous, without the need for clarification. ‘Slums as living spaces’ highlights the conditions of citizens in slums, and their experiences in their specific lived slum contexts. Second, by highlighting the embedded relation between slums and slum dwellers, the connection between various SDH elements could be delineated, and how these connections might lead (in)directly and/or together to the development of NCDs and their risk factors in slums. The RCA highlights the non-linear and multivariate causal links amongst identified NCD risk factors in slum communities. Making these pathways explicit may inspire researchers or policy makers to formulate interventions that assess the deduced causal relations experimentally, with the concomitant aim of tackling NCDs.

As the analysis took into account the complex reality of slum conditions, it allows for the possibility of developing policies and programmes that are more aligned to slum dwellers’ real health risks. For example, whilst empirical research has established that a poorly educated woman in the slums is more likely to develop CVD [36], it can miss that her low level of education further limits her decision-making power, which is solely tied to fulfilling household chores such as securing water for the family, cooking, cleaning and caring for the children. The interplay of these factors leaves her with a small window of opportunity for growth [57]. Thus, in targeting her health risks, the greater empowerment of women must be central to any health or education policy. Noteworthy examples include the alternative learning system (ALS) developed in the Philippines that caters for girls from slums who have dropped out of school [58], or the use of education coupons, which encourage parents to send their daughters to school [38]. NCDs in slums are thus not solely about physiological and individual propensities, but also about social structures (e.g. seclusion of women, the abusive attitude of teachers, low walkability, high costs of public transport) and how society, as a whole, manages the issues of people living in slums. Figure 3 indicates that policies stimulating economic production or educational output are not sufficient to address NCDs amongst slum residents, whilst Figure 4 shows that although poorly educated Indian men in slums are highly mobile, they are also more vulnerable to CVD due to their higher exposure to air and noise pollutants.

Based on the RCA, the following actions are recommended. First, the lack of clean water in slums is a broad issue requiring significant amounts of investment from national and local governments and private companies, which may take a long time to address. In the short term, however, relevant providers and municipalities should collaborate on a policy that ensures regular water deliveries of sufficient volume to slum communities with no plumbed water. This will eradicate the vast lengths of time spent in long lines for obtaining water and minimise water access irregularities. Second, as education is the ticket to ‘freedom’, the education of slum children, especially girls, is critical. Aside from an ALS for school drop-outs or education coupons for female students’ parents, a reward system for teachers of slum children could be implemented to raise the quality of teaching. The aim of the reward system is to instil greater self-esteem amongst educators of slum children, which could be realised through increased monetary benefits or chances for faster promotion or scholarship [59]. Third, for physical activity, activity spaces or hubs could be created in slums which would be used for yoga and meditation by slum dwellers. Alongside this, sports events could be organised with various non-governmental organisations, churches, local government and schools to encourage more vigorous forms of recreational physical activities. Finally, regarding public transportation, discount schemes that reduce the costs of transportation should be enhanced, whilst both local and national governments look for ways to establish a walkable and bike-friendly environment. The analsysis shows, however, that gender considerations must be integrated into the planning of transportation projects.

The strength of the research is based on its review of both quantitative and qualitative data, which show the experience of slum dwellers and their responses to their conditions. By triangulating these publications, it provides a more robust view of the issues surrounding NCDs from slum residents’ perspective. This did lead to the omission of some quantitative literature reporting well-established NCD risk factors, such as alcohol or tobacco use, which weren’t substantiated with qualitative data to determine their relation to other risk factors. Since the literature was gathered from various fields, the study is integrative and holistic in its approach. Nevertheless, the study is limited by the small amount of available literature on the issue. Further corroboration of the findings is necessary to facilitate a better understanding of NCDs and their risk factors in slums.

## Conclusion

This study contributes to analysing the literature on NCDs in slums for policy. It adopted a systems framing of the SDH model, combining quantitative associations of slum populations with qualitative data, to identify the dynamics of NCD risk in slums. Making the relations between NCD social determinants explicit is useful for identifying the most promising options for policy makers and local governments. This contributes to a greater understanding of the interactions between slums, their residents and their health, which could lead to more integrated approaches in tackling NCDs.

## Paper context

Non-communicable diseases (NCDs) are prevalent in slums, but there is limited knowledge on how they manifest. The Social Determinants of Health (SDH) account for the association between NCDs and socio-economic conditions, but knowing which to target through policy is operationally complex. Using root cause analysis, this paper traces the causal pathways of NCDs in slums identified by previous literature. The findings are valuable in the development of feasible policies and programmes targeting slum dwellers’ actual health needs.
